# Development of an In Vitro Screening Platform for the Identification of Partial PPARγ Agonists as a Source for Antidiabetic Lead Compounds

**DOI:** 10.3390/molecules23102431

**Published:** 2018-09-22

**Authors:** Lars Porskjær Christensen, Rime Bahij El-Houri

**Affiliations:** 1 Department of Chemistry and Bioscience, Faculty of Engineering and Science, Aalborg University, Fredrik Bajers Vej 7H, 9220 Aalborg Ø, Denmark; 2 Department of Chemical Engineering, Biotechnology and Environmental Technology, University of Southern Denmark, Campusvej 55, 5230 Odense M, Denmark; rbeh@kbm.sdu.dk

**Keywords:** type 2 diabetes, screening platform, partial PPARγ agonists, adipocytes, insulin, glucose uptake, PPARγ transactivation, adipocyte differentiation, in silico, plant extracts.

## Abstract

Type 2 diabetes (T2D) is a metabolic disorder where insulin-sensitive tissues show reduced sensitivity towards insulin and a decreased glucose uptake (GU), which leads to hyperglycaemia. Peroxisome proliferator-activated receptor (PPAR)γ plays an important role in lipid and glucose homeostasis and is one of the targets in the discovery of drugs against T2D. Activation of PPARγ by agonists leads to a conformational change in the ligand-binding domain, a process that alters the transcription of several target genes involved in glucose and lipid metabolism. Depending on the ligands, they can induce different sets of genes that depends of their recruitment of coactivators. The activation of PPARγ by full agonists such as the thiazolidinediones leads to improved insulin sensitivity but also to severe side effects probably due to their behavior as full agonists. Partial PPARγ agonists are compounds with diminished agonist efficacy compared to full agonist that may exhibit the same antidiabetic effect as full agonists without inducing the same magnitude of side effects. In this review, we describe a screening platform for the identification of partial PPARγ agonists from plant extracts that could be promising lead compounds for the development of antidiabetic drugs. The screening platform includes a series of in vitro bioassays, such as GU in adipocytes, PPARγ-mediated transactivation, adipocyte differentiation and gene expression as well as in silico docking for partial PPARγ agonism.

## 1. Introduction

In 2015, it was estimated that more than 415 million adults globally had diabetes mellitus, of which over 90% suffered from type 2 diabetes (T2D), and this number is projected to rise to around 642 million by 2040 [1]. Insulin resistance plays an essential role in the development of T2D, and is characterized by glucose intolerance resulting in elevated fasting glucose. Insulin resistance is mediated by high circulating levels of free fatty acids and by the release of certain hormones and inflammatory cytokines from adipose tissue, which impair insulin signaling in insulin sensitive tissues [2,3]. The hormones adiponectin and leptin are, however, important metabolic regulators that are crucial for maintaining a normal level of insulin sensitivity. Adiponectin increases the catabolism of free fatty acids and leptin increases energy expenditure leading to a reduction in insulin resistance. Insulin resistance is the prediabetic state where insulin sensitive tissues such as muscles and fat show reduced insulin sensitivity and a decrease in glucose uptake (GU). The prediabetic state is initially counteracted by an increased release of insulin from pancreatic β-cells to maintain glucose homeostasis. Eventually, the β-cells fail to sustain a sufficient insulin production resulting in hyperglycemia and T2D [2,3].

Patients suffering from T2D are treated with life-style changes including exercise and diet restriction, insulin and/or oral anti-hyperglycemic drugs. Anti-hyperglycemic drugs such as sulfonylureas and meglitinides (e.g., repaglinide) increase insulin secretion, biguanides (e.g., metformin) increase insulin sensitivity (insulin sensitizers), α-glucosidase inhibitors (e.g., acarbose and miglitol) work by preventing the digestion of starch and other carbohydrates in the small intestine and the thiazolidinediones (TZDs) such as rosiglitazone (Rosi) and pioglitazone are efficient insulin-sensitizing drugs [4,5,6,7]. TZDs act by activating peroxisome proliferator-activated receptor (PPAR)γ [6,7], which belong to a group of nuclear receptor proteins of ligand-inducible transcription factors regulating the expression of many genes. In mammals, there are beside PPARγ also PPARα and PPARβ/δ. PPARs consist of distinct functional domains including an N-terminal transactivation domain, a highly conserved DNA-binding domain (DBD), and a C-terminal ligand-binding domain (LBD) that contains a ligand-dependent transactivation function. The DBD anchors the PPARs to their binding sites on the DNA template. PPARs form obligate heterodimers with retinoid X receptor (RXR), which then binds to specific regions on the DNA of target genes. These specific DNA regions are termed PPAR response elements (PPREs). In the absence of a ligand, high-affinity complexes are formed between the inactive PPAR/RXR heterodimers and corepressor molecules. Upon ligand binding to the PPAR/RXR heterodimer, a conformational change in the LBD leads to release of the corepressor and binding of a coactivator resulting in expression of the target gene. PPARs control the expression of networks of genes involved in adipogenesis, lipid metabolism, inflammation, and maintenance of metabolic homeostasis [8,9,10,11,12,13]. *PPARα*, is expressed in kidneys, liver, heart and skeletal muscles as well as adipose tissue, where it is a major activator of fatty acid oxidation pathways and is the target of hypolipidemic drugs [12,14,15,16]. PPARβ/δ shares similar functions with PPARα, and is ubiquitously expressed and has a key role in fatty acid oxidation in skeletal muscle, liver and heart, and appears to be an important regulator of energy expenditure, and glucose and lipid metabolism [14,15,16]. PPARγ exists in two isoforms PPARγ1 and PPARγ2. *PPARγ1* is expressed in several tissues, including the lower intestines, macrophages, and adipose tissue, whereas *PPARγ2* is predominantly expressed in adipose tissue. The latter is the isoform of PPARγ, which is the focus of this review.

PPARγ is involved in many physiological processes and in particularly in the regulation of insulin sensitivity, inflammation, fatty acid storage, and glucose metabolism; hence, PPARγ represents an interesting pharmacological target being able to alleviate several of the underlying pathologies of T2D [6,9,10,12,13,15]. Activation of PPARγ leads to differential recruitment of coactivators and subsequent modulation of PPARγ activity. This process alters the transcription of several target genes involved in carbohydrate and lipid metabolism resulting in for example facilitation of GU and lipid uptake, decrease in free fatty acid levels and amelioration of insulin resistance [11,17,18,19,20,21,22]. Common dietary fatty acids such as oleic, linoleic and linolenic acids as well as endogenous prostanoids and phospholipids are ligands of PPARγ [11,23,24,25]. Other types of natural products that have been shown to be agonists of PPARγ are flavonoids, stilbenes, neolignans, amorfrutins, polyacetylenes, alkamides, sesquiterpene lactones, diterpenoids and triterpenoids [13,26,27,28,29,30]. Synthetic ligands of PPARγ as for example the TZDs are known to cause severe side effects such as increased water retention, oedema, weight gain, heart enlargement, and hepatotoxicity [30,31,32,33,34]; consequently, many TZDs drugs have been withdrawn from the market. The unwanted side effects of TZDs have been associated with their behavior as full agonists of PPARγ [7,11,30]. By contrast, partial PPARγ agonists are compounds with diminished agonist efficacy that maintain the insulin-sensitizing effect but usually without inducing the same magnitude of side effects as observed for full agonists [7,11]. In silico docking studies have revealed that full and partial agonists have different binding modes in the LBD of PPARγ [13,18,35,36,37,38]. This may explain why full and partial agonists recruit different sets of coactivators, and exhibits different pharmacological activities [13,18,39]. The search for PPARγ ligands with improved mechanisms of action is therefore an important objective to discover new promising antidiabetic compounds.

Plants have been used for centuries in the treatment of diabetes and are considered as a source for antidiabetic natural products [40,41,42,43]. The aim of this review is to introduce an in vitro screening platform for the identification of potential antidiabetic partial PPARγ agonists from complex plant extracts. The screening platform has for example been used to identify promising antidiabetic alkamides and polyacetylenes from purple coneflower (*Echinacea purpurea* (L.) Moench, Asteraceae) and carrots (*Daucus carota* L., Apiaceae), respectively, and how this has been done will be described.

## 2. Screening Platform

**Figure 1:**
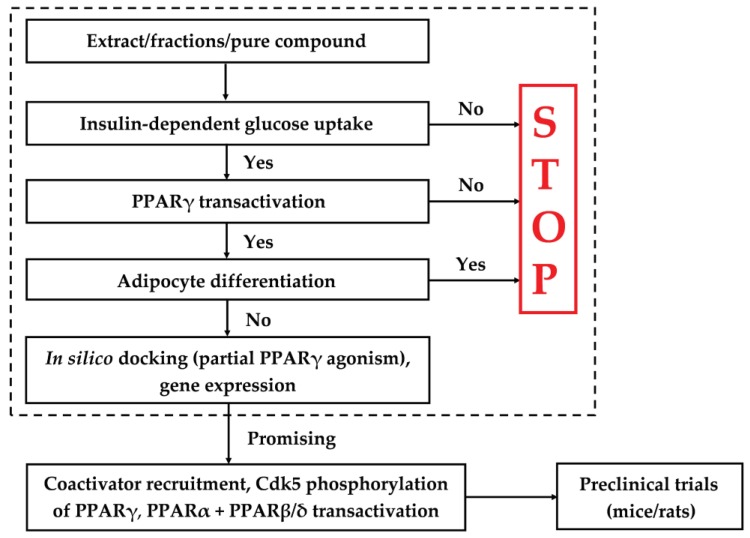
Screening platform for the identification of partial PPARγ agonists with potential antidiabetic properties. The most essential part of the screening platform is indicated in the dotted area.

The screening platform consists of several different bioassays as well as in silico docking for verification of partial PPARγ agonism, with the experimental strategy outlined in Figure 1.

The first step consist of a bioassay, which tests extracts, fractions and/or purified natural products (≈ test sample) for insulin-dependent GU in adipocytes. If a significant effect on GU is observed then testing is continued, otherwise it is stopped. In the next step, the ability of the test sample to activate PPARγ compared to a positive control (e.g., Rosi) is determined. In addition, the adipogenic potential of the test sample is determined in an adipocyte differentiation bioassay. If the test sample shows significantly lower activation of PPARγ and no significant stimulation of adipocyte differentiation compared to the positive control, then the test sample demonstrates promising antidiabetic effect that may be due to partial PPARγ agonism, and testing continues (Figure 1). Expression of genes involved in adipogenesis, lipogenesis, lipolysis, transportation and absorption of glucose may provide important information about the mechanisms of action of a test sample. If the test sample is a natural product with a known chemical structure, its partial PPARγ activity is investigated by in silico docking. A test sample that passes through all steps in the platform may contain natural products that may be antidiabetic lead compounds (Figure 1). In Figure 1, additional tests are also indicated, such as coactivator recruitment assays, testing for PPARα and PPAR β/δ transactivation as well as preclinical studies in rodents. PPARγ agonists that activate PPARα or PPARβ/δ are dual agonists. Agonists acting on all three PPAR receptors are pan agonists. Activation of PPARα and/or PPAR β/δ may result in the improvement of lipid profile, reduced adiposity and insulin sensitivity [44,45]. These additional tests are important to elucidate the overall activity profile of a promising antidiabetic lead compound, but will not be discussed in this review.

Extracts from numerous medicinal and food plants have been investigated for their potential antidiabetic effects [13,40,41,46]. For example, it has been shown that lipophilic extracts of roots of *E. purpurea* and carrots have promising antidiabetic effects, although these plants have not been used in traditional medicine for the treatment of diabetes. We have together with colleagues recently used the screening platform to characterize the potential antidiabetic compounds from these plant species [47,48], and this will be described in the following sections.

## 3. Insulin-Dependent and Basal GU

Insulin plays an essential role in glucose homeostasis by increasing storage or utilization of glucose by regulating the transport of glucose into the cell. Glucose transport into cells is catalyzed by several transport protein isoforms of which glucose transporter type 4 (Glut4), specifically facilitate glucose transport into insulin-sensitive tissue. Glut4 is insulin-dependent, and plays a key role in GU. In non-stimulated cells, the majority of Glut4 is found within intracellular storage vesicles. Upon stimulation by insulin, Glut4 redistributes and is incorporated into the plasma membrane of the cell where it mediates GU. Thus, insulin increases GU by increasing the concentration of Glut4 at the plasma membrane rather than enhancing the intrinsic activity of this transporter [49,50,51,52]. PPARγ activation by TZDs has been shown to increase the expression and translocation of Glut4 as well as the insulin-independent transporter type 1 (Glut1) to the cell surface. Consequently, PPARγ activation promote insulin-dependent and basal GU in adipocytes and muscle cells by upregulating gene expression for glucose transporters [31,53,54].

T2D is associated with a state of chronic inflammation in adipose tissue that secrete elevated levels of proinflammatory cytokines (e.g., TNFα, interleukin-6, and resistin), which all promotes insulin resistance [10,16,55,56,57]. Agonists of PPARγ have been shown to inhibit the expression of proinflammatory cytokines and to increase the plasma concentrations of the hormone adiponectin [10,16,30,58,59]. Adiponectin is positively associated with insulin sensitivity through increased fatty acid oxidation and inhibition of hepatic glucose production [59,60]. Adiponectin is present in relative high concentrations in plasma, but lower in obese subjects compared to lean subjects [61,62]. Reduced levels of adiponectin are in particular found in patients diagnosed with T2D [59,62]. A significant increase in insulin-dependent GU in adipocytes of a test sample is strong evidence that it contains compound(s) that positively affect insulin trafficking.

### 3.1. Identification of Potential Antidiabetic Compounds from Plants with Effect on Insulin-Dependent GU

To study insulin-dependent GU in adipocytes two approaches can be used. Firstly, one can investigate the effect at different concentrations at a fixed insulin concentration, which is usually 10 nM insulin corresponding to the insulin concentration in human healthy subjects. The concentration is normally in the range between 1–100 µg/mL for extracts or fractions, and for pure natural products from 0.1–30 µM to avoid cytotoxic effects and because higher concentrations indicates too low antidiabetic efficacy. Another approach is to investigate insulin-dependent GU at a fixed concentration, which is typically between 1–100 µg/mL for extracts/fractions and from 0.1–30 µM for pure natural products, at different insulin concentrations (e.g., at 3, 10, 30 and 100 nM) or with no insulin to determine basal GU [47,48].

#### 3.1.1. Alkamides in the Roots of *E. purpurea* Show an Effect on Insulin-Dependent GU

**Figure 2:**
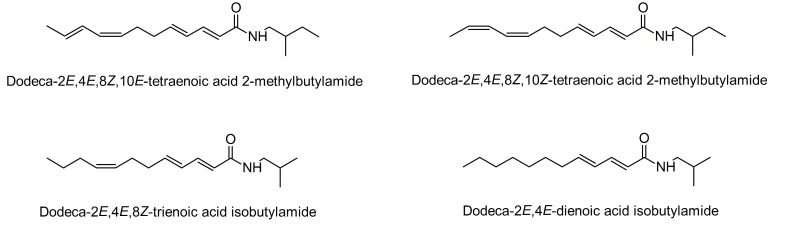
Chemical structures of alkamides isolated from an active fraction of a DCM root extract of *Echinacea purpurea* that demonstrated significant insulin-dependent GU activity.

In a recent study by Kotowska et al. [47], it was demonstrated that a dichloromethane (DCM) extract (100 µg/mL) of the roots of *E. purpurea* increased basal GU 2-fold in mature 3T3-L1 adipocytes compared to the vehicle (DMSO). Further, the GU was enhanced in the presence of 3 and 10 nM of insulin. These results suggests that this extract contain natural products that increases insulin sensitivity in 3T3-L1 adipocytes [47]. Separation of the DCM extract by normal-phase flash column chromatography (flash CC) resulted in nine fractions (A–I). Fractions A and D increased insulin-dependent GU with the latter being the most active fraction. Investigation of the active fractions by high-performance liquid chromatography−diode array detection−tandem mass spectrometry (HPLC-DAD-MS/MS) revealed that fraction A contained the PPARγ agonist α-linolenic acid as a major constituent. Fraction D contained an inseparable mixture of the novel isomeric alkamides dodeca-2*E*,4*E*,8*Z*,10*E/Z-*tetraenoic acid 2-methylbutylamides in a 1:1 ratio as shown by NMR spectroscopy [47], as well as the known alkamides dodeca-2*E*,4*E*,8*Z*-trienoic acid isobutylamide and dodeca-2*E*,4*E*-dienoic acid isobutylamide, which were isolated by semi-preparative HPLC (Figure 2).

**Figure 3:**
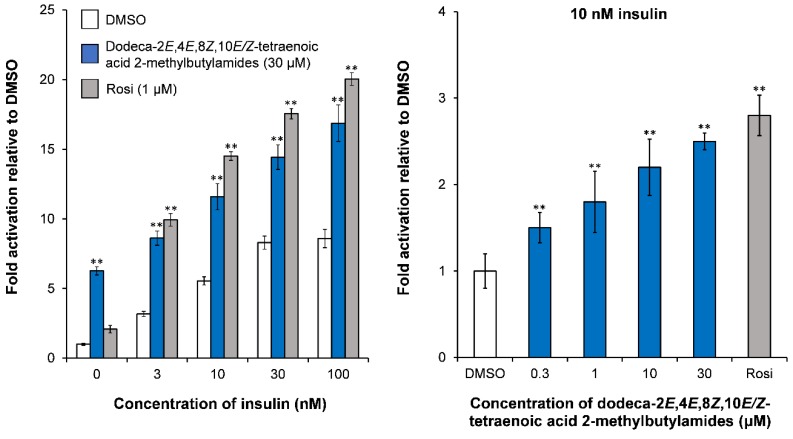
Effect of dodeca-2*E*,4*E*,8*Z*,10*E*/*Z*-tetraenoic acid 2-methylbutylamides on insulin-dependent GU at 30 μM in mature 3T3-L1 adipocytes and dose-dependent effect at 10 nM insulin. The results are normalized to the vehicle (DMSO), which was set to 1. Rosi (1 μM) was the positive control. All values are expressed as a mean ± SD of three independent experiments in triplicates. ** *p* < 0.001 indicates significance relative to DMSO.

The isomeric dodeca-2*E*,4*E*,8*Z*,10*E/Z-*tetraenoic acid 2-methylbutylamides at 30 μM resulted in a significant increase in basal GU compared to the vehicle (DMSO) and the positive control Rosi (Figure 3). These alkamides also significantly stimulated insulin-dependent GU (Figure 3). On the other hand dodeca-2*E*,4*E*,8*Z*-trienoic acid isobutylamide and dodeca-2*E*,4*E*-dienoic acid isobutylamide showed only a weak increase of basal GU and insulin-dependent GU in mature 3T3-L1 adipocytes at 30 μM [47]. Thus dodeca-2*E*,4*E*,8*Z*,10*E/Z-*tetraenoic acid 2-methylbutylamides seem to be the main contributors to the observed potential antidiabetic effect of the DCM extract of *E. purpurea* roots and were selected for further studies in the screening platform.

#### 3.1.2. Polyacetylenes from Carrot Roots Show an Effect on Insulin-Dependent GU

**Figure 4:**
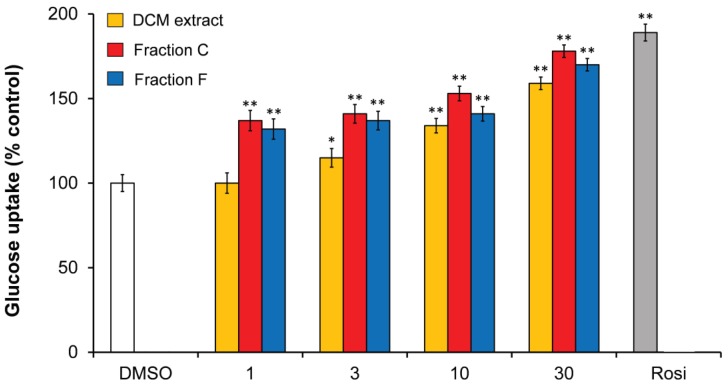
Effect of a DCM extract of carrot roots and active fractions (C and F) on insulin-dependent GU in mature 3T3-L1 adipocytes at 10 nM insulin, relative to 0.1 % DMSO (vehicle, 100%) and the positive control Rosi (1 μM). All values are expressed as a mean ± SD of three independent experiments in triplicates. * *p* < 0.01, ** *p* < 0.001, indicate significance relative to the vehicle.

**Figure 5:**
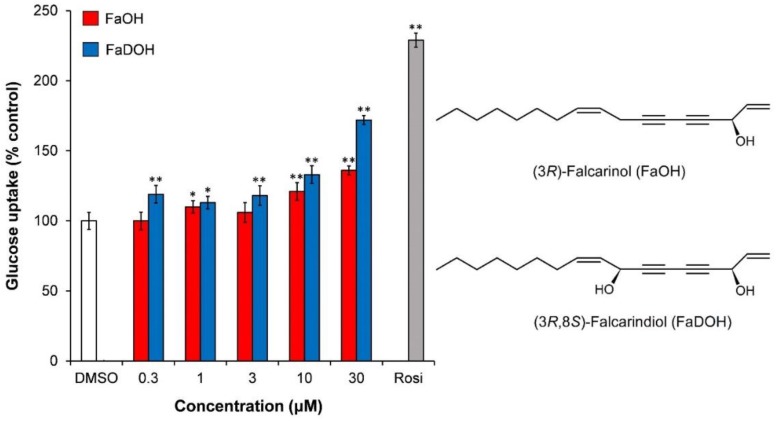
Effect of FaOH and FaDOH on insulin-dependent GU in mature 3T3-L1 adipocytes relative to 0.1% DMSO (vehicle, 100%). Insulin concentration was 10 nM, and the positive control was Rosi (1 μM). All values are expressed as a mean ± SD of three independent experiments in triplicates. * *p* < 0.01, ** *p* < 0.001, indicate significance relative to the vehicle.

In a study by El-Houri et al. [48], it has recently been shown that the DCM extract of carrot roots in concentrations between 1–30 µg/mL stimulated insulin-dependent GU in 3T3-L1 adipocytes at 10 nM insulin in a dose dependent manner (Figure 4). Fractionation of the extract by normal-phase flash CC resulted in 10 fractions (A–J) of which fraction C and F showed significant stimulation of insulin-dependent GU relative to the vehicle (0.1% DMSO). Fraction C and F increased insulin-dependent GU in a dose-dependent relationship (Figure 4). Fraction C contained mainly the polyacetylene (3*R*)-falcarinol (FaOH) while fraction F contained mainly (3*R*,8*S*)-falcarindiol (FaDOH) as shown by HPLC-DAD-MS/MS and other chemical analysis (Figure 5) [48]. 

Purified FaOH and FaDOH showed an increased insulin-dependent GU in the concentration range between 0.3–30 µM with FaDOH being more potent than FaOH at all concentrations tested (Figure 5).

## 4. PPARγ Transactivation Assay

Recruitment of coactivators to the PPARγ-RXR complex in response to different ligands leads to major differences in the transactivation of target genes. Thus, the challenge is to find ligands with an optimal biological profile, i.e., ligands that recruit coactivators resulting in antidiabetic effects with no or reduced adverse effects compared to full PPARγ agonists.

Partial PPARγ agonists are compounds that activate PPARγ weaker than full agonists, and in addition may induce recruitment of beneficial coactivators with regard to controlling glucose homeostasis, energy expenditure, insulin-dependent GU, and adipogenesis. Numerous coactivators have been identified and includes the steroid receptor coactivator (SRC) family of coactivators, PPARγ coactivator-1 (PGC-1) family of coactivators, CBP (CREB binding protein), and TRAP220 of the mediator complex [11,20,55,63,64,65] of which some coactivators result in beneficial effects and others in adverse effects [66]. The steroid receptor coactivator-1 (SRC-1) regulates key metabolic pathways, including glucose homeostasis. Furthermore, *SRC-1*-deficient mice have been shown to have reduced energy expenditure and to be susceptible to obesity; thus SRC-1 is a beneficial coactivator [66,67,68]. PPARγ coactivator 1-α (PGC-1α) is a key coactivator for regulating glucose metabolism and whole body energy expenditure and is also a beneficial coactivator [66,69]. On the other hand, the TRAP220 coactivator of the mediator complex has been shown to interact with PPARγ and to stimulate adipogenesis, resulting in expression of genes associated with lipid storage and weight gain [70]. The coactivator transcriptional mediator/intermediary factor 2 (TIF2)/SRC-2 also promotes fat accumulation in adipocytes [67] and therefore TRAP220 and TIF2 are considered as adverse coactivators. Finally, PPARγ agonists may also recruit repressors and an example of such a ligand-dependent transcriptional repressor is receptor interacting protein (RIP) 140, which represses genes involved in energy expenditure [71]. A desired partial PPARγ agonist profile should therefore result in recruitment of coactivators that improves glucose metabolism and energy expenditure and not induce adipogenesis. A test sample that in the screening platform significantly improves insulin- stimulated GU and exhibit significantly less activation of PPARγ compared to TZDs, indicates that it possess the characteristics of a partial PPARγ agonist recruiting beneficial coactivators.

### 4.1. Identification of Potential Partial PPARγ Agonists from Plants

Plant extracts have high hit rates when tested for PPARγ activity [40,41,46], because they often contain common PPARγ activators such as polyunsaturated fatty acids. Consequently, PPARγ activity assays cannot stand alone in the search for promising antidiabetic natural products but need to be supported by additional information (Figure 1). PPARγ ligand activity of a test sample is usually determined using a PPARγ transactivation bioassay such as a luciferase reporter gene cell-based assay. In case of pure compounds, a competitive PPARγ receptor-binding assay in agonist mode using a time-resolved fluorescence resonance energy transfer (TR-FRET) assay with purified PPARγ protein is also often used in combination with the PPARγ transactivation bioassay [13]. The binding assay with a receptor protein is one of the most direct approaches to confirm a potential binding and/or interaction of a ligand with PPARγ. Application of a receptor-binding assay is, however, not sufficient to guarantee that a compound can act also in cells in vivo, due to its inability to penetrate cellular membranes, transportation out of cells mediated by membrane efflux transporters or metabolic transformation to products that are not PPARγ ligands [13]. PPARγ transactivation bioassays may compensate for the pitfalls in the receptor-binding assay but these cellular models then have other disadvantages. For example, PPARγ activation determined in a luciferase reporter cell-based assay might be the result of indirect effects such as increase in PPARγ protein expression, activation of RXR etc. [13]. Despite the different weaknesses of the methods for the determination of PPARγ activity, they are relatively fast and provide important information on whether a compound is as a partial or full PPARγ agonist or an extract/fraction contains promising PPARγ activators.

#### 4.1.1. PPARγ Activity of Alkamides from *E. purpurea*

Dodeca-2*E*,4*E*,8*Z*,10*E*/*Z*-tetraenoic acid 2-methylbutylamides were shown to significantly activate PPARγ at a concentration of 30 μM compared to the vehicle DMSO, but the effect was weak compared to Rosi [47]. Furthermore, dodeca-2*E*,4*E*,8*Z*,10*E*/*Z*-tetraenoic acid 2-methylbutylamides and Rosi were shown to compete with a fluorescent pan PPAR agonist in a ligand binding assay indicating a common binding site in the PPARγ LBD [47]. The results indicates that these alkamides are partial PPARγ agonists, which was confirmed by in silico docking (see Section 6.1).

#### 4.1.2. PPARγ Activity of FaOH and FaDOH from Carrots

FaDOH was shown to significantly activate PPARγ 3-fold, at the highest tested concentration (30 μM) compared to the vehicle (0.1% DMSO) in a PPARγ transactivation bioassay. FaOH showed a weak but significant 1.6-fold activation of PPARγ at 10 μM, but not at 3 µM and 30 μM. PPARγ activation of FaOH and FaDOH was significant lower compared to the positive control Rosi [48]. The results indicates that FaOH and FaDOH are partial PPARγ agonists, which was confirmed by in silico docking studies (see Section 6.1). For FaDOH, this is in accordance with a study of Atanasov et al. [72] who showed that FaDOH binds to purified human PPARγ and activates PPARγ-dependent reporter gene expression as a partial agonist between 1–30 µM, and antagonizes the effect of Rosi.

## 5. Adipocyte Differentiation

PPARγ play a key role in the regulation of lipid storage and control of fatty acid metabolism and is considered a master regulator of adipocyte differentiation [9,64,73,74]. Another transcription factor that is strongly involved in adipogenesis is cytidine-cytidine-adenosine-adenosine-thymidine (CCAAT)/enhancer-binding protein α (C/EBPα). However, it is unclear how PPARγ and C/EBPα function cooperatively during the adipocyte differentiation process. An important protein involved in adipogenesis is adipocyte protein 2 (aP2), which is a key mediator of intracellular transport and metabolism of fatty acids. *aP2* gene is highly expressed in adipocytes and macrophages and plays an important role in the development of insulin resistance and atherosclerosis. The expression of aP2 is highly regulated during adipocyte differentiation by PPARγ and C/EBPα [75,76]. Stearoyl-coenzyme A desaturase (SCD1) is a key enzyme in the control of lipid metabolism and is rate limiting for the conversion of saturated fatty acids to monounsaturated fatty acids and thus the formation of triglycerides and other lipids. *SCD1* gene is highly expressed in adipose tissue [77,78]. PPARγ upregulation of *SCD1* leads to increased lipogenesis and elevated levels of SCD1 is associated with obesity. This is also in accordance with the fact that *SCD1*-deficient or knockout mice are protected from obesity and show increased insulin sensitivity [79,80]. Therefore, overexpression of *SCD1* in humans may be involved in the development of T2D, hypertriglyceridemia, and atherosclerosis.

Adipose triglyceride lipase (ATGL) and hormone-sensitive lipase (HSL) are the major key enzymes involved in the breakdown of triglycerides to fatty acid derivatives and their activity are regulated by insulin. The free fatty acid derivatives released during lipolysis can serve as intrinsic ligands for PPARγ and can impair insulin-signaling [81,82]; hence, the regulation of *ATGL* and *HSL* are important in relation to obesity, T2D, and related metabolic disorders.

### 5.1. Adipocyte Differentiation Bioassays and Gene Expression

PPARγ play a central role in the differentiation of preadipocytes into mature adipocytes. The process of cell differentiation by which preadipocytes become adipocytes is complex and involves genes, which are a part of the insulin-signaling cascade, thereby improving insulin sensitivity [83]. The critical window for ligand-dependent induction of adipocyte differentiation of 3T3-L1 cells is days 0 to 4 after induction of differentiation. To induce differentiation of 3T3-L1 preadipocytes into mature adipocytes, a cocktail consisting of the synthetic glucocorticoid dexamethasone, 1-methyl-3-isobutylxanthine, and insulin (MDI protocol) is often used [40,41,47,48,84].

PPARγ stimulates the production of small insulin-sensitive adipocytes but also plays an important role in regulating lipid metabolism in mature adipocytes by increasing fatty acid trapping and storage of lipids in adipose tissue. This results in lowering of circulating free fatty acids in liver and muscles as well as modifying secretion of hormones from adipose tissue, all factors known to improve insulin sensitivity. The insulin sensitizing effects of full PPARγ agonist are linked to their ability to increase adipogenesis and to shift the energy balance toward storage and redistribution of fat from visceral to subcutaneous adipose tissue as well as to increase the population of small adipocytes and simultaneously decrease the population of large adipocytes [8,11,15,30]. This may explain the insulin-sensitizing effects of TZDs but also one of their major side effects, which is increase in whole-body adiposity (weight gain). Thus, in the search for potential antidiabetic natural products that activate PPARγ, it is essential that they do not significantly stimulate adipocyte differentiation and fat accumulation.

The adipogenic potential of a test sample, in this screening platform is determined in preadipocytes and/or in mature adipocytes using the MDI protocol and Oil Red O to stain the triglycerides. Together with gene expression studies in preadipocyes and mature adipocytes, it gives useful information of potential antidiabetic effects of a test sample [27,28,40,41,47,48]. With regard to fat accumulation, the worm *Caenorhabditis elegans* has been shown to serve as an excellent in vivo model for fast screening for fat accumulation, which can provide useful information about energy homeostasis and fat storage pathways in a whole organism [40,85]. However, this in vivo model is not an essential part of the screening platform and will not be discussed in this review. 

### 5.2. Investigation of Adipocyte Differentiation of Dodeca-2E,4E,8Z,10E/Z-Tetraenoic Acid 2-Methyl-butylamides

**Figure 6:**
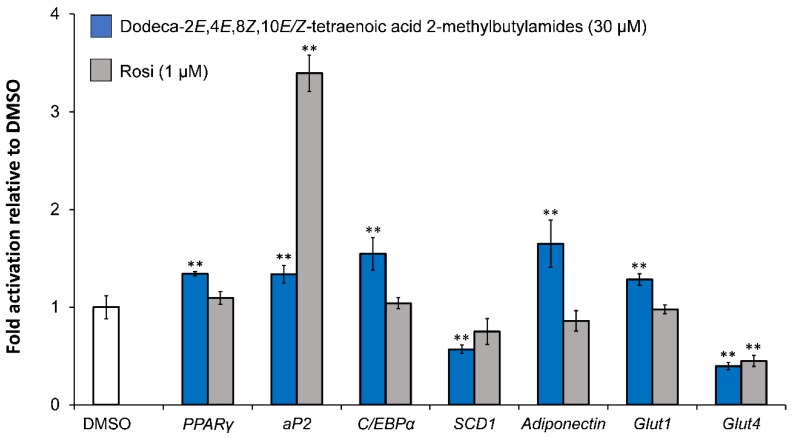
Effect of dodeca-2*E*,4*E*,8*Z*,10*E*/*Z*-tetraenoic acid 2-methylbutylamides (30 μM) on gene expression of proteins involved in adipogenesis (PPARγ, C/EBPα, aP2), glucose transport (Glut1, Glut4), lipogenesis (SCD1), and adipokines (adiponectin) in mature 3T3-L1 cells. Rosi (1 μM) was the positive control. All values are normalized to the vehicle DMSO and are expressed as a mean ± SD of three independent experiments in triplicates. ** *p* < 0.001 indicates significance relative to DMSO.

Kotowska et al. [47] investigated the effect of dodeca-2*E*,4*E*,8*Z*,10*E*/*Z*-tetraenoic acid 2-methylbutylamides on adipogenesis, glucose transport, lipogenesis, and adipokines in the early stages of adipocyte differentiation in mature 3T3-L1 cells treated with 30 μM of the alkamides. Dodeca-2*E*,4*E*,8*Z*,10*E*/*Z*-tetraenoic acid 2-methylbutylamides were shown to increase the gene expression of key markers for adipogenesis (PPARγ, aP2, C/EBPα) as well as adiponectin and Glut1, whereas the gene expression for Glut4 and SCD1 was significantly downregulated (Figure 6). 

The downregulation of *SCD1* gene expression may result in decreased lipogenesis and a small size of lipid droplets [86]. *Glut1* is expressed in both preadipocytes and mature adipocytes, whereas *Glut4* is expressed only in mature adipocytes. The upregulation of *Glut1* gene expression is in accordance with the enhanced basal GU observed for dodeca-2*E*,4*E*,8*Z*,10*E*/*Z*-tetraenoic acid 2-methylbutylamides, whereas the observed effect on insulin-dependent GU of these alkamides (Figure 3) appears not to be related to the gene expression of glucose transporters [47]. Adiponectin also plays an important role in mediating GU in adipocytes and was significantly upregulated by dodeca-2*E*,4*E*,8*Z*,10*E*/*Z*-tetraenoic acid 2-methylbutylamides, which suggests that the adipocytes might be insulin sensitive [87] in accordance with their effect on insulin-dependent GU.

### 5.3. Investigation of Adipocyte Differentiation of FaOH and FaDOH

**Figure 7:**
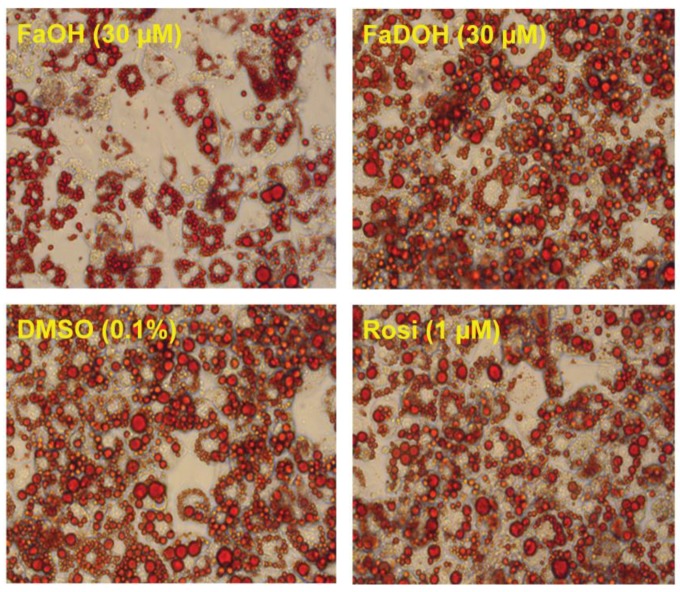
Effect of FaOH (30 μM), FaDOH (30 μM), vehicle (0.1% DMSO) and Rosi (1 μM), respectively, on adipocyte differentiation (MDI protocol) in 3T3-L1 preadipocyte. Cells were stained with Oil Red O on day 8.

FaOH impair adipocyte differentiation as shown in 3T3-L1 preadipocytes whereas this is not the case for FaDOH (Figure 7) [48]. The impairment of adipocyte differentiation by FaOH may reflect competition between endogenous activators of PPARγ needed for induction of adipocyte differentiation in combination with the weak activation of PPARγ by FaOH.

**Figure 8:**
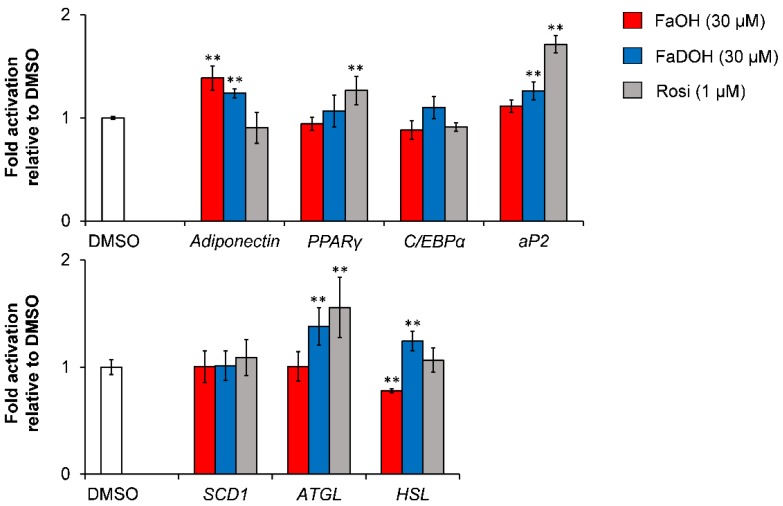
Effect of FaOH and FaDOH on gene expression of key proteins involved in adipogenesis (PPARγ, C/EBPα, aP2), lipogenesis (SCD1), lipolysis (ATGL, HSL) and the adipokine adiponectin in mature 3T3-L1 cells. Rosi was the positive control. All values are normalized to the vehicle 0.1% DMSO and are expressed as a mean ± SD of three independent experiments in triplicates. ** *p* < 0.001 indicates significance relative to 0.1% DMSO.

El-Houri et al. [48] observed no upregulation of the key markers of adipogenesis (PPARγ, C/EBPα) for FaOH and FaDOH (Figure 8). However, a significant upregulation of the gene expression of *aP2* was found in response to treatment with FaDOH, whereas FaOH was found not to have an effect on *aP2* gene expression (Figure 8). These results are in accordance with the impairment of adipocyte differentiation observed for FaOH and both polyacetylenes being weak activators of PPARγ. The significant upregulation of *aP2* by FaDOH clearly indicates that it is more potent with regard to a potential antidiabetic effect and has a higher efficacy in relation to PPARγ activation than FaOH. This is also in accordance with other investigations of this type of polyacetylenes [13,72].

FaOH and FaDOH did not affect *SCD1* gene expression significantly in accordance with the results from the adipocyte differentiation assay (Figure 7). However, a significant upregulation of the gene expression of *ATGL* and *HSL* was observed for FaDOH (Figure 8). Expression of these genes has been shown to contribute to the increase of plasma fatty acids that affect insulin sensitivity [88]. In contrast, FaOH significantly downregulated the expression of the *HSL* gene and had no significant effect on *ATGL* gene expression (Figure 8).

The differences between FaOH and FaDOH on expression of *ATGL* and *HSL* as well as on adipocyte differentiation, indicates that these polyacetylenes have distinct mechanisms of action in adipocytes. This was supported by in silico docking studies that showed that FaOH and FaDOH had different affinities to the PPARγ LBD (see Section 6.1). Thus, it could be interesting to investigate the effect of FaOH and FaDOH on a wider range of transcription factors involved in adipogenesis, recruitment of PPARγ coactivators, and glucose transporters in order to elucidate their possible mechanisms of action and evaluate their potential antidiabetic effects in preclinical trials.

## 6. In Silico Screening for Identification of PPARγ Agonists

In silico screening is an important technique for selecting promising antidiabetic compounds for experimental testing in vitro and in vivo as well as to verify and understand the binding modes of ligands towards nuclear receptors. Docking and structure-based 3D pharmacophores are the most used approaches within in silico screening. Docking is used to investigate the binding modes of a ligand at a protein-binding site whereas structure-based 3D pharmacophores describe protein-ligand interactions by various chemical features that can then be used for virtual screening [18,37,38]. Different docking methods exist and structural details for the binding of an agonist to the PPARγ LBD are available for over 100 receptor-ligand crystal structures of which several have been solved for different natural products [18]. Investigation of these crystallographic structures have revealed two specific binding modes in the PPARγ LBD, which correspond to full and partial agonists, respectively.

The LBD consists of 13 α-helices labelled H1–H12 and H2’, as well as one β-sheet region. The ligand-binding pocket of PPARγ LBD is located in the centre of the LBD and is a large Y-shaped ligand-binding cavity, consisting of three binding pockets (arm I–III) of which each pocket have different properties and binding preferences. Arm III is the entrance arm that branches off into arm I and arm II. Arm I is extended toward H12 of the ligand-dependent activation domain whereas arm II is situated between H3 and the β-sheet. Arm I is the polar cavity of the PPARγ LBD, whereas arm II and the interior of arm III are mainly hydrophobic [11,13,18]. Full agonists occupy arm I and form hydrogen bonds with the amino acids Ser289, His323, His449, and Tyr473. These interactions stabilize H12 and are mainly responsible for the PPARγ activity of full agonists. In addition, full agonists also interact with arm II through a hydrophobic tail but these interactions appears to be of minor importance in relation to the transactivation activity of PPARγ [11,13,18]. Partial agonists interact mainly with amino acids in arm III forming a hydrogen bond to Ser342, but interact also with arm II through several hydrophobic interactions [11,13,18,47,48]. This binding mode of partial PPARγ agonists causes a lower degree of H12 stabilization and an increase in the stabilization of H3, which affects the recruitment of coactivators and decreases the transactivation activity of PPARγ. This explains why the PPARγ transactivation activity is lower for partial agonists compared to full agonists but does not explain why agonists with different PPARγ transactivation activities may have similar insulin-sensitizing activities. The antidiabetic efficacy of different ligands does not only correlate with the ligand-binding affinity but also their ability to inhibit phosphorylation of PPARγ by cyclin-dependent kinase 5 (Cdk5) at Ser273 in PPARγ. The latter prevent the expression of target genes involved in lipid and glucose homeostasis, and seem to play a key role in the insulin--sensitizing effect of PPARγ agonists [18,31,89,90]. TZDs have been shown to inhibit the Cdk5-mediated phosphorylation of PPARγ in adipose tissue and the same is true with PPARγ ligands with poor agonistic activity but with potent antidiabetic effects in vivo. The inhibition of Ser273 phosphorylation by ligands does not seem to depend on the degree of classical agonist action [13,31,89,90]. The antidiabetic effect of full and partial agonists of PPARγ may therefore in part be explained by the inhibition of PPARγ phosphorylation. However, the classical agonist action seem to explain the side effects of full agonists. Thus, an effective PPARγ agonist should have a weak transactivation activity, but high phosphorylation inhibitory activity of PPARγ [13,18,89,90]. Testing for inhibition of Cdk5-mediated PPARγ phosphorylation is not a part of the screening platform but should be considered as an additional test aiming at elucidating the overall activity profile of promising antidiabetic natural products that act as partial PPARγ agonists.

### 6.1. In Silico Docking for Investigation of Partial PPARγ Agonism of Natural Products

**Figure 9:**
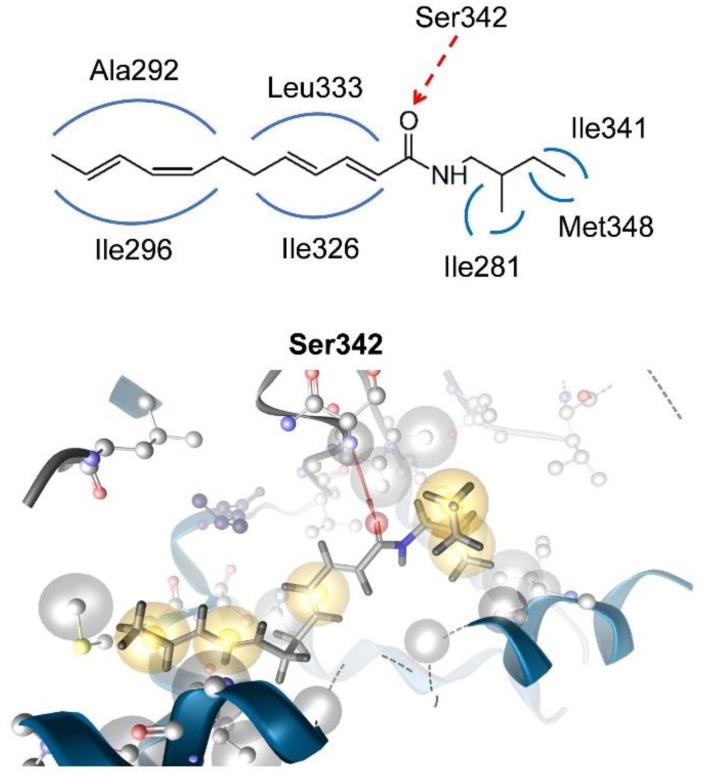
The predicted binding modes of dodeca-2*E*,4*E*,8*Z*,10*E*-tetraenoic acid 2-methylbutylamide to the PPARγ LBD illustrated in a 2D-model (**top**) and 3D-model (**bottom**). The chemical interaction pattern is the same for dodeca-2*E*,4*E*,8*Z*,10*Z*-tetraenoic acid 2-methylbutylamide. Binding modes to the LBD in the 2D-model are color-coded: red dashed arrow = hydrogen bond; blue line = hydrophobic interactions.

**Figure 10:**
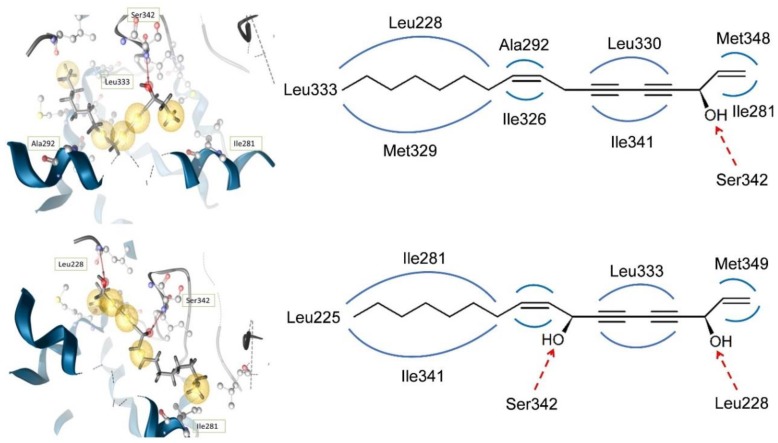
The predicted binding modes of FaOH and FaDOH to the PPARγ LBD illustrated in a 2D-model (**top**) and a 3D-model (**bottom**). Binding modes to the LBD in the 2D-model are color-coded: red dashed arrow = hydrogen bond; blue line = hydrophobic interactions.

Knowing the exact chemical structure of a PPARγ agonist, it is possible to perform in silico docking of the agonist in the PPARγ LBD and determine whether the binding mode of the ligand resembles that of a full or a partial PPARγ agonist. Kotowska et al. [47] and El-Houri et al. [48] used the docking method GOLD version 5.1 and default parameters (GoldScore, 100% search efficiency) to determine partial PPARγ agonism of dodeca-2*E*,4*E*,8*Z*,10*E*/*Z*-tetraenoic acid 2-methylbutylamides and FaOH and FaDOH, respectively (Figure 9 and Figure 10). The active site was determined by selecting all residues within a radius of 6 Å of the co-crystalized ligand for human PPARγ. After docking, the compounds were minimized using LigandScout software and the best docking poses for the ligands were selected by developing a 3D pharmacophore [18,47,48].

In silico docking studies of the dodeca-2*E*,4*E*,8*Z*,10*E*/*Z*-tetraenoic acid 2-methylbutylamides into the PPARγ LBD predicted binding modes to one hydrogen bond with the amino acid Ser342 and hydrophobic interactions with several amino acids from arms II and III (Figure 9) [47]. In addition, no hydrogen bond interaction between dodeca-2*E*,4*E*,8*Z*,10*E*/*Z*-tetraenoic acid 2-methylbutylamides and amino acids from arm I was predicted. Thus, the docking mode of dodeca-2*E*,4*E*,8*Z*,10*E*/*Z*-tetraenoic acid 2-methylbutylamides into the PPARγ LBD combined with the weak activation of PPARγ as well as the results of the competitive PPARγ binding assay, suggests that these compounds are acting as PPARγ partial agonists.

In silico docking of FaOH and FaDOH into the PPARγ LBD revealed that the different PPARγ activities of these polyacetylenes to some extent can be explained by their binding modes to the PPARγ LBD. The best docking pose for FaOH established a hydrogen bond between the amino acid Ser342 and the hydroxyl group at C-3. For FaDOH the best docking pose predicted a hydrogen bond between the hydroxyl group at C-3 and the amino acid Leu228 and between the hydroxyl group at C-8 and Ser342.

In addition, in silico docking established several hydrophobic interactions between the LBD and FaOH and FaDOH, respectively (Figure 10) [48]. The extra hydrogen bond of FaDOH to the LBD of PPARγ compared to FaOH may explain why FaDOH is a more effective activator of PPARγ than FaOH. The predicted binding modes of FaOH and FaDOH with arms II and III, clearly indicates that these natural products are partial PPARγ agonists. For FaDOH this is in accordance with previous investigations using another docking model [13,72].

The PPARγ activating properties of alkamides and polyacetylenes as well as closely related endogenous and/or dietary PPARγ ligands appears to depend on the length of their aliphatic chain and functional groups, and thus on their interactions with the LBD [27,47]. However, the large cavity of the PPARγ LBD, and thus the broad specificity of this receptor, makes the prediction of PPARγ activity of small molecules difficult. Therefore, in silico docking can only be considered as an important tool to confirm that a natural product may act as a partial PPARγ agonist and to give a reasonable explanation for its partial PPARγ agonism.

## 7. Conclusions

The screening platform presented in this review has been developed based on our experience with bioassay-guided fractionation of plant extracts for the isolation and characterization of promising antidiabetic compounds. The screening platform has shown to be rather efficient in isolating potential antidiabetic partial PPARγ agonists from complex plant extracts as demonstrated by the isolation of potential antidiabetic alkamides and polyacetylenes from the roots of *E. purpurea* and carrots, respectively. A bioassay-guided fractionation approach of the lipophilic extract of the roots of *E. purpurea* using an insulin-dependent GU bioassay resulted in the isolation of an inseparable mixture of two new C_12_-isomeric alkamides demonstrating promising antidiabetic effects by significantly enhancing basal GU and insulin-dependent GU in adipocytes and exhibiting the characteristics of PPARγ partial agonists. Similarly, the screening platform has been used to identify the potential antidiabetic polyacetylenes FaOH and FaDOH from the lipophilic extracts of carrots. The polyacetylenes enhanced GU in adipocytes in a dose-dependent manner and displayed also the characteristics of PPARγ partial agonists. FaOH inhibited adipocyte differentiation as evident by gene expression studies and Oil Red O staining, whereas this was not the case for FaDOH. This indicates that these polyacetylenes may have distinct mechanisms of action. In silico docking experiments with FaOH and FaDOH, respectively, into the PPARγ LBD revealed that these polyacetylenes have different binding modes that may result in the recruitment of different beneficial PPARγ coactivators and thus confirm that FaOH and FaDOH may have distinct mechanisms of action. Coactovator recruitment is not an essential part of the screening platform but are important bioassays in order to provide further information on the mechanisms of action of the antidiabetic effects of purified partial PPARγ agonists. In addition, some partial PPARγ agonists may function as dual PPAR agonists or pan PPAR agonists and/or inhibit phosphyrolation of PPARγ, which are all important factors for elucidating the mechanisms of action of partial PPARγ agonists and hence evaluate their antidiabetic potential and possible side effects, before entering preclinical trials.

In conclusion, the screening platform presented in this review represents an effective method for the identification of potential antidiabetic partial PPARγ agonists from complex extracts that can turn out to be lead compounds for developing drugs for the prevention and/or treatment of T2D.
